# *catena*-Poly[(dichloridozinc)-μ-1-{4-[(1*H*-imidazol-1-yl)meth­yl]benz­yl}-1*H*-imidazole-κ^2^ *N* ^3^:*N* ^3′^]

**DOI:** 10.1107/S1600536812015395

**Published:** 2012-04-18

**Authors:** Cheng Wang, Bo Wen, Zhi-Yao Sun, Peng-Fei Yan, Jin-Sheng Gao

**Affiliations:** a Key Laboratory of Functional Inorganic Material Chemistry, Ministry of Education, Heilongjiang University, Harbin 150080, People’s Republic of China; b Engineering Research Center of Pesticides of Heilongjiang University, Heilongjiang University, Harbin 150050, People’s Republic of China

## Abstract

The asymmetric unit of the title compound, [ZnCl_2_(C_14_H_14_N_4_)]_*n*_, contains a Zn^II^ ion situated on a twofold rotation axis and one-half of a 1-{4-[(1*H*-imidazol-1-yl)meth­yl]benz­yl}-1*H*-imidazole (*L*) ligand with the benzene ring situated on an inversion center. The Zn^II^ ion is coordinated by two chloride anions and two N atoms from two *L* ligands in a distorted tetra­hedral geometry. The *L* ligands bridge ZnCl_2_ fragments into polymeric chains parallel to [20-1].

## Related literature

For the synthesis of the ligand, see: Yang *et al.* (2006 ▶).
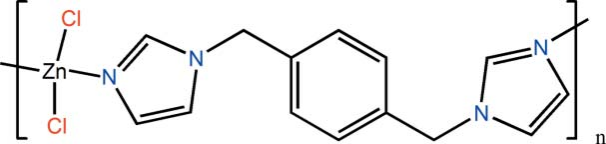


## Experimental

### 

#### Crystal data

[ZnCl_2_(C_14_H_14_N_4_)]
*M* *_r_* = 374.56
Orthorhombic, 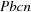

*a* = 11.327 (2) Å
*b* = 10.207 (2) Å
*c* = 14.452 (3) Å
*V* = 1670.8 (6) Å^3^
*Z* = 4
Mo *K*α radiation
μ = 1.79 mm^−1^
*T* = 293 K
0.58 × 0.55 × 0.49 mm

#### Data collection

Rigaku R-AXIS RAPID diffractometer
Absorption correction: multi-scan (*ABSCOR*; Higashi, 1995 ▶) *T*_min_ = 0.421, *T*_max_ = 0.477
15231 measured reflections
1916 independent reflections
1718 reflections with *I* > 2σ(*I*)
*R*_int_ = 0.022

#### Refinement

*R*[*F* ^2^ > 2σ(*F* ^2^)] = 0.023
*wR*(*F* ^2^) = 0.063
*S* = 1.08
1916 reflections
96 parameters
H-atom parameters constrained
Δρ_max_ = 0.20 e Å^−3^
Δρ_min_ = −0.29 e Å^−3^

### 

Data collection: *RAPID-AUTO* (Rigaku, 1998 ▶); cell refinement: *RAPID-AUTO*; data reduction: *CrystalClear* (Rigaku/MSC, 2002 ▶); program(s) used to solve structure: *SHELXS97* (Sheldrick, 2008 ▶); program(s) used to refine structure: *SHELXL97* (Sheldrick, 2008 ▶); molecular graphics: *SHELXTL* (Sheldrick, 2008 ▶); software used to prepare material for publication: *SHELXL97*.

## Supplementary Material

Crystal structure: contains datablock(s) global. DOI: 10.1107/S1600536812015395/cv5275sup1.cif

Additional supplementary materials:  crystallographic information; 3D view; checkCIF report

## Figures and Tables

**Fig. 1.**
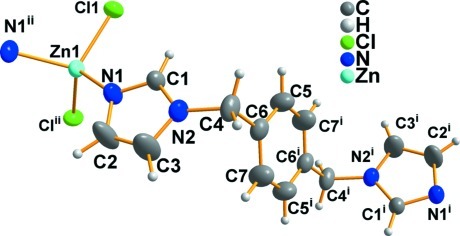
The molecular structure of the title compound, showing the atomic numbering and displacement ellipsoids at the 50% probability level [symmetry codes: (i) 1-x, -y, 1-z; (ii) 2-x, y, 0.5-z].

**Fig. 2.**
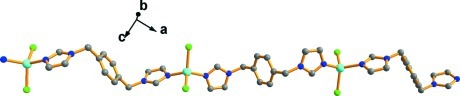
A portion of the polymeric chain in the title compound. H atoms omitted for clarity.

## supplementary crystallographic information

### Comment

The synthesis and characterization of coordination networks based on the idea of self-assembly of specifically designed building blocks has been an area of rapid growth in recent years. Herein, we report the title compound constructed by 1-[4-[(1*H*-imidazol-1-yl)methyl]benzyl] -1*H*-imidazole and ZnCl_2_.

The asymmetric unit of the title compound, [ZnCl_2_*L*]_n_ (*L* = 1-[4-[(1*H*-imidazol-1-yl)methyl]benzyl] -1*H*-imidazole, C_14_H_14_N_4_), contains a Zn^II^ ion situated on a twofold rotational axis and one-half ligand *L* with the benzene ring situated on an inversion center. Each Zn^II^ ion is coordinated by two chlorido anions and two N atoms from two ligands *L* in a distorted tetrahedral geometry (Figure 1). Ligands *L* bridge ZnCl_2_ fragments into polymeric chains in [20-1] (Figure 2).

### Experimental

The 1-[4-[(1*H*-imidazol-1-yl)methyl]benzyl] -1*H*-imidazole was synthesized following the reference method (Yang *et al.*, 2006). Synthesis of the title compound: ligand (0.120 g, 0.5 mmol) and ZnCl_2_ (0.080 g, 0.5 mmol) were dissolved in a mixed solution of 4 mL ethanol and 4 mL water. After stirring, the suspension was sealed in a 18 mL Teflon-lined autoclave and heated at 140 °C for 5 days. After slow cooling to room temperature, colorless block crystals were filtered and washed with distilled water (52% yield based on Zn).

### Refinement

C-bound H atoms were placed in calculated positions and treated as riding on their parent atoms, with C—H = 0.93 Å (aromatic); C—H = 0.97 Å (methylene), and with *U*_iso_(H) = 1.2*U*_eq_(C).

### Crystal data

**Table:**

[ZnCl_2_(C_14_H_14_N_4_)]	*F*(000) = 760
*M**_r_* = 374.56	*D*_x_ = 1.489 Mg m^−^^3^
Orthorhombic, *P**b**c**n*	Mo *K*α radiation, λ = 0.71073 Å
Hall symbol: -P 2n 2ab	Cell parameters from 13075 reflections
*a* = 11.327 (2) Å	θ = 3.0–27.5°
*b* = 10.207 (2) Å	µ = 1.79 mm^−^^1^
*c* = 14.452 (3) Å	*T* = 293 K
*V* = 1670.8 (6) Å^3^	Block, colourless
*Z* = 4	0.58 × 0.55 × 0.49 mm

### Data collection

**Table:**

Rigaku R-AXIS RAPID diffractometer	1916 independent reflections
Radiation source: fine-focus sealed tube	1718 reflections with *I* > 2σ(*I*)
Graphite monochromator	*R*_int_ = 0.022
ω scan	θ_max_ = 27.5°, θ_min_ = 3.0°
Absorption correction: multi-scan (*ABSCOR*; Higashi, 1995)	*h* = −14→14
*T*_min_ = 0.421, *T*_max_ = 0.477	*k* = −13→13
15231 measured reflections	*l* = −18→16

### Refinement

**Table:**

Refinement on *F*^2^	Primary atom site location: structure-invariant direct methods
Least-squares matrix: full	Secondary atom site location: difference Fourier map
*R*[*F*^2^ > 2σ(*F*^2^)] = 0.023	Hydrogen site location: inferred from neighbouring sites
*wR*(*F*^2^) = 0.063	H-atom parameters constrained
*S* = 1.08	*w* = 1/[σ^2^(*F*_o_^2^) + (0.0339*P*)^2^ + 0.4633*P*] where *P* = (*F*_o_^2^ + 2*F*_c_^2^)/3
1916 reflections	(Δ/σ)_max_ = 0.001
96 parameters	Δρ_max_ = 0.20 e Å^−^^3^
0 restraints	Δρ_min_ = −0.29 e Å^−^^3^

### Special details

**Table:**

Geometry. All esds (except the esd in the dihedral angle between two l.s. planes) are estimated using the full covariance matrix. The cell esds are taken into account individually in the estimation of esds in distances, angles and torsion angles; correlations between esds in cell parameters are only used when they are defined by crystal symmetry. An approximate (isotropic) treatment of cell esds is used for estimating esds involving l.s. planes.
Refinement. Refinement of F^2^ against ALL reflections. The weighted R-factor wR and goodness of fit S are based on F^2^, conventional R-factors R are based on F, with F set to zero for negative F^2^. The threshold expression of F^2^ > 2sigma(F^2^) is used only for calculating R-factors(gt) etc. and is not relevant to the choice of reflections for refinement. R-factors based on F^2^ are statistically about twice as large as those based on F, and R- factors based on ALL data will be even larger.

### Fractional atomic coordinates and isotropic or equivalent isotropic displacement parameters (Å^2^)

**Table:**

	*x*	*y*	*z*	*U*_iso_*/*U*_eq_	
C1	0.83519 (13)	0.15817 (15)	0.39823 (10)	0.0350 (3)	
H1	0.8723	0.1028	0.4402	0.042*	
C2	0.7938 (2)	0.2662 (3)	0.27631 (15)	0.0754 (8)	
H2	0.7976	0.3004	0.2168	0.090*	
C3	0.7085 (2)	0.2926 (3)	0.33876 (15)	0.0733 (7)	
H3	0.6436	0.3472	0.3304	0.088*	
C4	0.66639 (15)	0.21680 (18)	0.50158 (12)	0.0458 (4)	
H4A	0.6237	0.2983	0.5099	0.055*	
H4B	0.7195	0.2065	0.5537	0.055*	
C5	0.59983 (16)	−0.0048 (2)	0.55434 (14)	0.0540 (5)	
H5	0.6667	−0.0088	0.5916	0.065*	
C6	0.57941 (13)	0.10417 (16)	0.50083 (11)	0.0396 (4)	
C7	0.47871 (17)	0.1088 (2)	0.44701 (16)	0.0556 (5)	
H7	0.4635	0.1826	0.4112	0.067*	
Cl1	1.08205 (3)	−0.03773 (4)	0.36609 (3)	0.04045 (11)	
N1	0.87372 (11)	0.18141 (14)	0.31401 (9)	0.0391 (3)	
N2	0.73601 (11)	0.22419 (13)	0.41575 (9)	0.0374 (3)	
Zn1	1.0000	0.07951 (2)	0.2500	0.03065 (9)	

### Atomic displacement parameters (Å^2^)

**Table:**

	*U*^11^	*U*^22^	*U*^33^	*U*^12^	*U*^13^	*U*^23^
C1	0.0346 (7)	0.0395 (8)	0.0308 (7)	0.0067 (6)	0.0040 (6)	0.0015 (6)
C2	0.0835 (16)	0.0979 (19)	0.0448 (10)	0.0500 (14)	0.0178 (10)	0.0316 (11)
C3	0.0737 (14)	0.0870 (16)	0.0592 (12)	0.0511 (13)	0.0165 (10)	0.0251 (11)
C4	0.0439 (9)	0.0499 (10)	0.0437 (9)	−0.0023 (7)	0.0163 (7)	−0.0125 (8)
C5	0.0408 (9)	0.0609 (12)	0.0603 (11)	−0.0004 (8)	−0.0097 (8)	0.0075 (10)
C6	0.0346 (8)	0.0439 (9)	0.0402 (8)	0.0035 (6)	0.0096 (6)	−0.0074 (7)
C7	0.0489 (10)	0.0507 (11)	0.0674 (13)	0.0018 (8)	−0.0055 (9)	0.0146 (10)
Cl1	0.0367 (2)	0.0478 (2)	0.0369 (2)	0.00320 (16)	−0.00610 (14)	0.00531 (16)
N1	0.0407 (7)	0.0447 (8)	0.0319 (6)	0.0120 (6)	0.0078 (5)	0.0048 (6)
N2	0.0360 (6)	0.0390 (7)	0.0371 (7)	0.0064 (5)	0.0079 (5)	−0.0017 (5)
Zn1	0.02814 (14)	0.03731 (15)	0.02650 (14)	0.000	0.00440 (8)	0.000

### Geometric parameters (Å, º)

**Table:**

C1—N1	1.3146 (19)	C4—H4B	0.9700
C1—N2	1.3343 (19)	C5—C6	1.375 (3)
C1—H1	0.9300	C5—C7^i^	1.384 (3)
C2—C3	1.350 (3)	C5—H5	0.9300
C2—N1	1.365 (2)	C6—C7	1.381 (3)
C2—H2	0.9300	C7—C5^i^	1.384 (3)
C3—N2	1.350 (2)	C7—H7	0.9300
C3—H3	0.9300	Cl1—Zn1	2.2606 (5)
C4—N2	1.4718 (19)	N1—Zn1	1.9959 (13)
C4—C6	1.514 (2)	Zn1—N1^ii^	1.9959 (13)
C4—H4A	0.9700	Zn1—Cl1^ii^	2.2606 (5)
			
N1—C1—N2	111.35 (14)	C5—C6—C7	118.89 (16)
N1—C1—H1	124.3	C5—C6—C4	120.07 (16)
N2—C1—H1	124.3	C7—C6—C4	121.04 (17)
C3—C2—N1	109.55 (17)	C6—C7—C5^i^	120.84 (19)
C3—C2—H2	125.2	C6—C7—H7	119.6
N1—C2—H2	125.2	C5^i^—C7—H7	119.6
C2—C3—N2	106.38 (16)	C1—N1—C2	105.29 (14)
C2—C3—H3	126.8	C1—N1—Zn1	124.96 (11)
N2—C3—H3	126.8	C2—N1—Zn1	128.33 (12)
N2—C4—C6	112.43 (13)	C1—N2—C3	107.42 (14)
N2—C4—H4A	109.1	C1—N2—C4	125.82 (14)
C6—C4—H4A	109.1	C3—N2—C4	126.67 (15)
N2—C4—H4B	109.1	N1^ii^—Zn1—N1	117.19 (8)
C6—C4—H4B	109.1	N1^ii^—Zn1—Cl1^ii^	103.09 (4)
H4A—C4—H4B	107.8	N1—Zn1—Cl1^ii^	108.98 (4)
C6—C5—C7^i^	120.26 (17)	N1^ii^—Zn1—Cl1	108.98 (4)
C6—C5—H5	119.9	N1—Zn1—Cl1	103.09 (4)
C7^i^—C5—H5	119.9	Cl1^ii^—Zn1—Cl1	116.08 (3)

Symmetry codes: (i) −*x*+1, −*y*, −*z*+1; (ii) −*x*+2, *y*, −*z*+1/2.
